# Single-epitope T cell–based vaccine protects against SARS-CoV-2 infection in a preclinical animal model

**DOI:** 10.1172/jci.insight.167306

**Published:** 2023-04-10

**Authors:** Takuya Tada, Ju-Yi Peng, Belinda M. Dcosta, Nathaniel R. Landau

**Affiliations:** Department of Microbiology, NYU Grossman School of Medicine, New York, New York, USA.

**Keywords:** COVID-19, Immunology, Dendritic cells, T cells

## Abstract

Currently authorized COVID-19 vaccines induce humoral and cellular responses to epitopes in the SARS-CoV-2 spike protein, though the relative roles of antibodies and T cells in protection are not well understood. To understand the role of vaccine-elicited T cell responses in protection, we established a T cell–only vaccine using a DC-targeted lentiviral vector expressing single CD8^+^ T cell epitopes of the viral nucleocapsid, spike, and ORF1. Immunization of angiotensin-converting enzyme 2–transgenic mice with ex vivo lentiviral vector–transduced DCs or by direct injection of the vector induced the proliferation of functional antigen-specific CD8^+^ T cells, resulting in a 3-log decrease in virus load upon live virus challenge that was effective against the ancestral virus and Omicron variants. The Pfizer/BNT162b2 vaccine was also protective in mice, but the antibodies elicited did not cross-react on the Omicron variants, suggesting that the protection was mediated by T cells. The studies suggest that the T cell response plays an important role in vaccine protection. The findings suggest that the incorporation of additional T cell epitopes into current vaccines would increase their effectiveness and broaden protection.

## Introduction

The emergency use authorized vaccines for SARS-CoV-2 have been highly effective at reducing rates of hospitalization and mortality but less effective at preventing infection (1, 2). Vaccines in current use are based on the viral spike (S) protein. The decreased effectiveness of the vaccines in preventing infection largely results from the rapidly changing S protein that has accumulated mutations in the receptor binding domain (RBD) and N-terminal domain that allow the virus to escape neutralization by vaccine-elicited antibodies (3). The vaccines also induce a T cell response to epitopes within the S protein (4–10), and these remain largely unmutated in the variants. The relative roles of the B and T cell responses in the protective effect of vaccination are not well understood. The decreased effectiveness of vaccine protection against the variants suggests an important role for antibodies in protection. Neutralizing antibody titers elicited by the parental S protein encoding mRNA vaccines were decreased by 5- to 30-fold against the Omicron variants (11–17). The current bivalent booster encoding BA.4/5 and parental S proteins elicits antibodies against the Omicron variants with titers that are 2- to 5-fold higher than that of the previous vaccines but that are still not equivalent to those against the ancestral virus (15, 18–23). While antibody titers elicited by the vaccines are decreased against the Omicron variants, they are highly effective at preventing severe disease (18). Continued protection against hospitalization and death may be the result of the T cell response, which is less subject to immunoevasion by mutations in the viral S protein (6, 8, 24). Furthermore, T cell immunity may cross-react with seasonal and other pandemic coronaviruses (6, 25–27).

Several lines of evidence suggest that CD8^+^ T cells play a role in mitigating COVID-19 disease severity and providing long-term immune protection (28–32). Mild COVID-19 disease is associated with robust CD8^+^ T cell reactivity to viral epitopes and rapid CD8^+^ T cell–mediated viral clearance (33). Selective decreases in CD8^+^, but not CD4^+^, T cell numbers are associated with an unfavorable prognosis and systemic inflammation (34). Depletion of CD8^+^ T cells from convalescent macaques decreased protective immunity against infection. The same was true for SARS-CoV-2–infected mice (35, 36). CD8^+^ T cell responses to both natural SARS-CoV-2 infection and vaccination are long-lasting (6, 32, 37). In individuals who recovered from SARS-CoV-1 infection, virus-specific IgG titers peaked 4 months postinfection and then declined after 1 year while antigen-specific CD8^+^ T cells persisted for at least 17 years (38).

Previous reports using mouse models have demonstrated the effectiveness of vaccine-elicited T cells against SARS-CoV-2 and identified the critical CD8^+^ T cell epitopes. Joag et al. showed that successive heterologous intramuscular (IM) or intranasal (IN) immunizations with a modified vaccinia virus Ankara adenoviral vector and DNA encoding the SARS-CoV-2 nucleocapsid (N) protein resulted in a robust CD8^+^ T cell response (39). A major determinant of the CD8 response was identified as the nucleocapsid peptide epitope N_219-227_ (LALLLLDRL), which is also an important epitope in the human CD8^+^ T cell response (39). Other epitopes found to induce robust CD8^+^ T cell responses in mice include N_105-113_ (SPRWYFYYL) (40), ORF1_1637–1646_ (TTDPSFLGRY) (41), and S_539-546_ (VNFNFNGL) epitopes (42, 43). A single intravenous (IV) injection or IN injection with the Ad-5-N adenoviral vector induced a T cell response that protected mice from lethal challenge with SARS-CoV-2 (44, 45). Ku et al. showed that an intraperitoneal followed by IN vaccination with a lentiviral vector expressing the S protein induced humoral and cellular responses that protected against SARS-CoV-2 infection in hamster and mouse models (46).

We previously reported on the use of a lentiviral vector–based vaccine to protect against lethal infection of mice with lymphocytic choriomeningitis virus (LCMV) (47, 48). The vectors expressed single CD8^+^ T cell epitopes or a combination of CD8^+^ and CD4^+^ T cell epitopes and coexpressed CD40L, which served to mature transduced dendritic cells (DCs) and induce the secretion of Th1 cytokines such as IL-12p70, TNF-α, and IL-6, resulting in potent T cell responses against the virus. Administration of the vectors either by ex vivo–transduced DCs or direct lentivirus injection elicited functional antiviral T cells that reduced virus loads by 3 orders of magnitude. Similar results were obtained with lentiviral vectors expressing a CD8^+^ T cell epitope from HIV in a humanized mouse model of HIV infection (49).

In this report, we further tested the effectiveness of the T cell responses in protecting against SARS-CoV-2 disease pathogenesis using lentiviral vectors that expressed single CD8^+^ T cell epitopes derived from the S, N, and ORF1 genes. The protective response was both rapid and independent of the humoral response as the vectors encoded no surface-exposed neutralizing epitopes. The injecting of ex vivo–transduced DCs primed highly functional, antigen-specific CD8^+^ T cells that decreased virus loads by 3 orders of magnitude. The effects of the vaccine were durable and equally protective against the ancestral D614G virus and Omicron variants. The findings highlight the efficacy of the CD8^+^ T cell responses in suppressing SARS-CoV-2 replication and suggest that the inclusion of additional T cell epitopes in current vaccines would add to their breadth and effectiveness.

## Results

### DC vaccination protects mice from SARS-CoV-2 infection.

**Figure 1:**
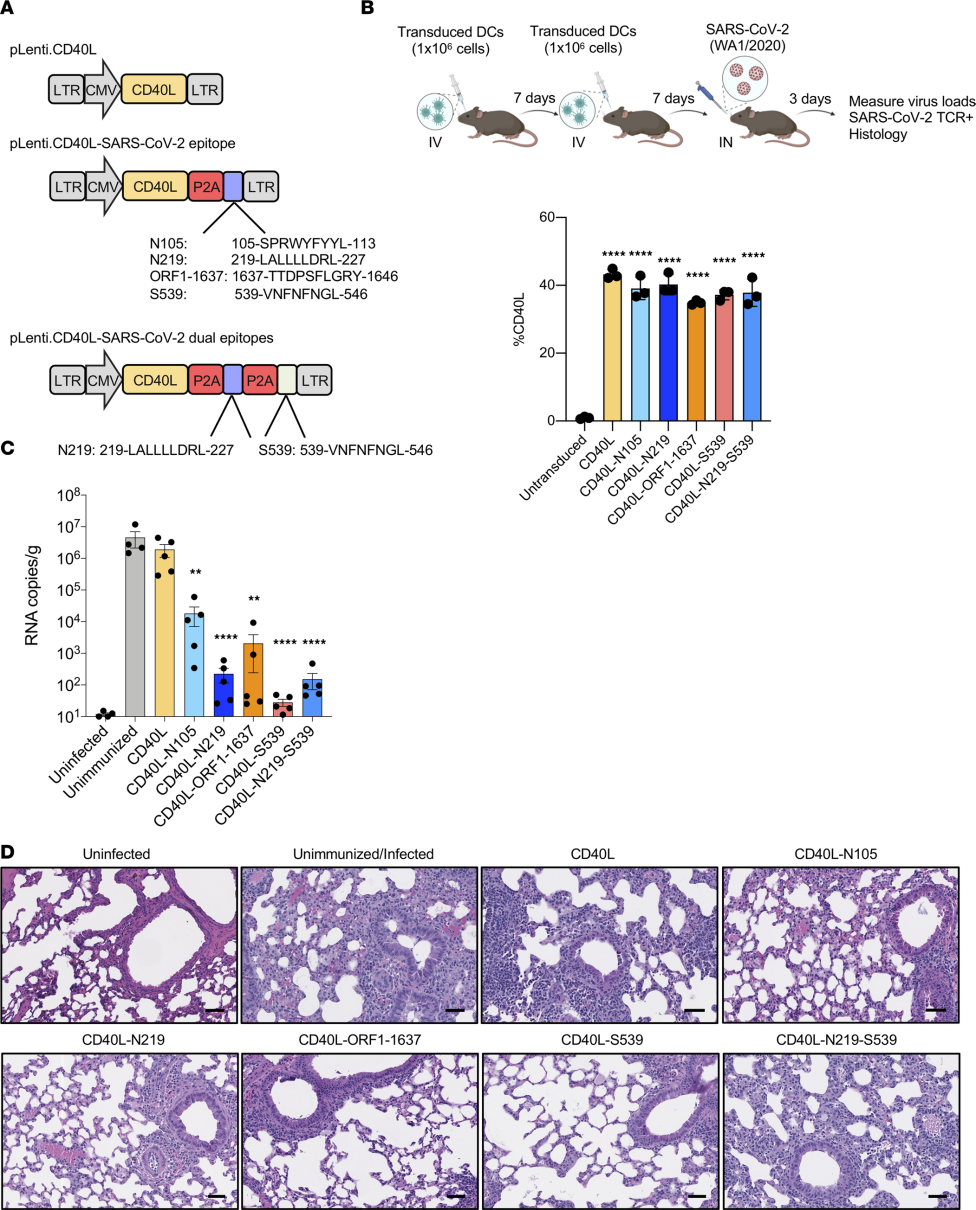
DC vaccine protects mice against SARS-CoV-2 infection. (**A**) Schematic of lentiviral vectors expressing murine CD40L and CD8^+^ T cell epitopes N_219-227_, N_105-113_, ORF1_1637-1646_, S_539-546_, and N_219-227_-S_539-546_. (**B**) The experimental scheme for testing vaccine protection is diagrammed. hACE2-KI mice were immunized by 2 IV injections of 1 × 10^6^ transduced DCs, 1 week apart (*n* = 6). The mice were challenged 1 week after the second immunization with 2 × 10^4^ PFU SARS-CoV-2 WA1/2020. SAMHD1-KO BMDCs were transduced with lentiviral vectors at MOI = 5, and CD40L expression was analyzed 3 dpi by flow cytometry (bottom). The experiment was done twice with similar results. hACE2-KI, human angiotensin-converting enzyme 2–knockin; dpi, days postinfection. (**C**) Viral subgenomic RNA in the lungs of the immunized mice was measured by RT-qPCR 3 days postchallenge. The *y* axis of the histograms shows the viral RNA copy numbers/g lung tissue. Statistical significance was determined by Kruskal-Wallis test with post hoc Dunn’s test. Confidence intervals are shown as the mean ± SD. (**P ≤ 0.01, ****P ≤ 0.0001.) The experiment was done 3 times with similar results. (**D**) Hematoxylin and eosin staining of lung sections from unimmunized and lentiviral vector–immunized mice (original magnification, 20×; scale bars: 50 μm). Each image is representative of 6 mice.

To test whether the T cell response alone would be sufficient to protect mice against SARS-CoV-2 infection, we generated lentiviral vectors that expressed CD8^+^ T cell epitopes recognized by mouse H2b. These included epitope N (N_105-113_: 105-SPRWYFYYL-113) (40), (N_219-227_: 219-LALLLLDRL-227) (39), ORF1 (ORF1_1637-1646_: 1637-TTDPSFLGRY-1646) (41), and S epitope (S_539-546_: 539-VNFNFNGL-546) (42, 43). Vectors were constructed that expressed single T cell epitopes (N_219-227_, ORF1_1637-1646_, or S_539-546_) and a vector that expressed both the N and S epitope (N_219-227_-S_539-546_). The epitopes were expressed as fusions to the carboxy terminal of CD40L, which served to induce DC activation and maturation, with an intervening 2A sequence of porcine teschovirus-1 (P2A) processing peptide. Fusion of the epitope to the carboxy terminal of CD40L has been found to promote efficient antigen presentation on class I MHC proteins (Figure 1A). A control vector expressed CD40L alone. We tested the vectors by the transduction of bone marrow–derived dendritic cells (BMDCs) of sterile alpha motif and HD-domain containing protein 1–knockout (SAMHD1-KO) mice, the cells of which are transduced by lentiviral vectors 3- to 4-fold more efficiently than wild-type (47). Transduction of the SAMHD1-KO BMDCs at an MOI of 5 resulted in 38%–40% of the cells expressing CD40L (Figure 1B).

To test whether the T cell epitope–expressing vectors would induce a protective immune response to SARS-CoV-2, hACE2-KI mice were injected with transduced DCs and 7 days later boosted by a second injection. Seven days after the second injection, the mice were challenged with SARS-CoV-2 by IN inoculation, and 3 dpi, virus loads in the lung were measured by reverse transcription quantitative PCR (RT-qPCR). Analysis of the viral RNA showed higher virus loads (2 × 10^6^ copies/g) in the unimmunized and CD40L-expressing DC–immunized mice. Immunization with vectors expressing the N_105-113_, N_219-227_, ORF1_1637-1646_, S_539-546_, and N_219-227_-S_539-546_ vectors decreased the virus load another 100- to 68,000-fold, with the largest decrease caused by the S_539-546_ epitope (Figure 1C). Analysis of the lungs by histopathological H&E staining showed that the lungs of infected but unimmunized mice had signs of interstitial pneumonia with thickened alveolar septa and inflammatory cell infiltration (Figure 1D). The lungs of mice immunized with control CD40L vector had a similar appearance. In contrast, the lungs of mice immunized with transduced DCs showed little sign of pneumonia and few infiltrating inflammatory cells.

**Figure 2:**
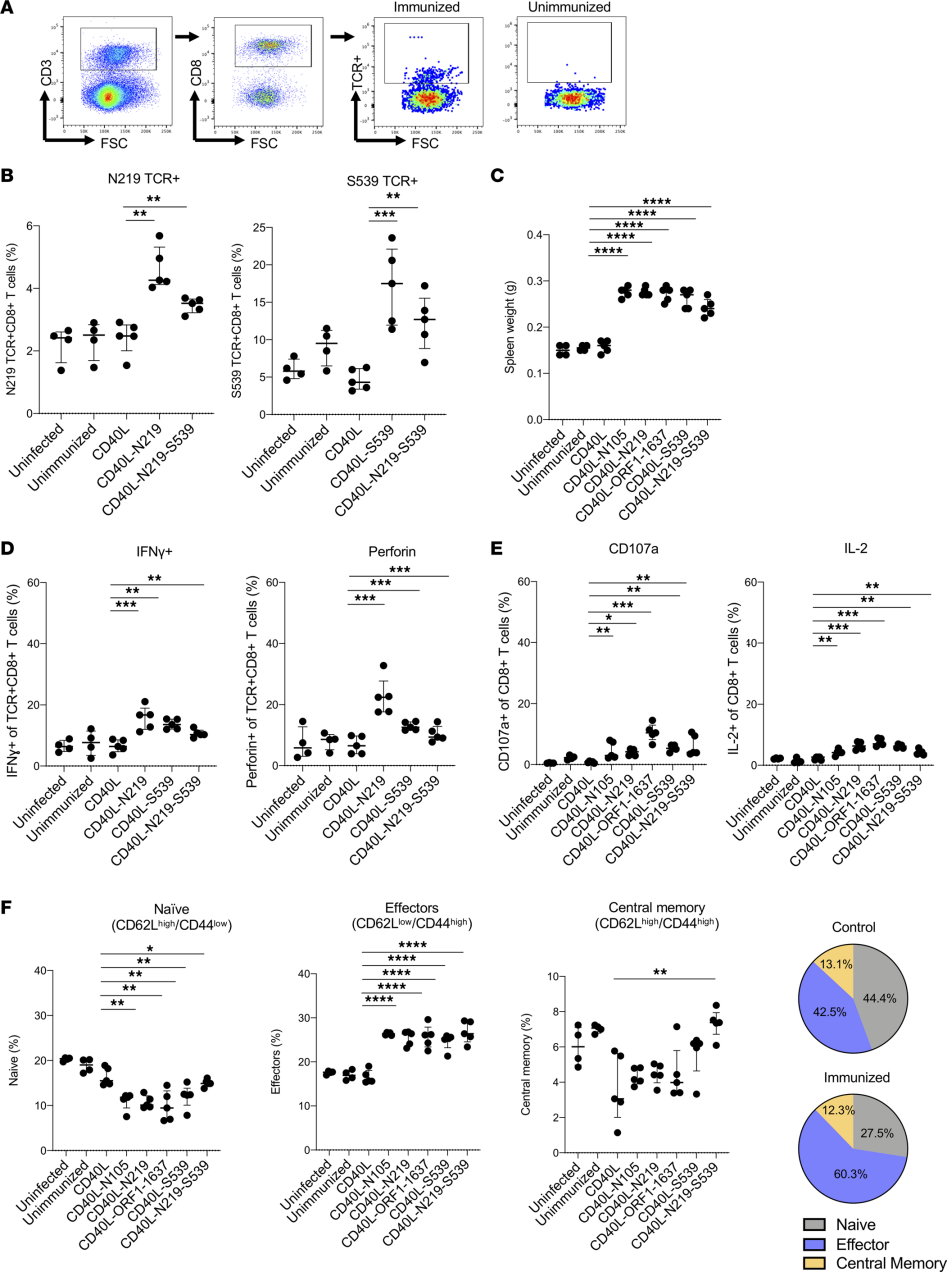
T cell response induced from lentivirus-based DC vaccine. (**A**) The gating scheme used in the analysis of the antigen-specific CD8^+^ T cells induced by vaccination is shown for a representative sample from a single mouse. Splenocytes from mice immunized with 2 injections of transduced DCs and then challenged with SARS-CoV-2 WA1/2020 or splenocytes from unimmunized mice and then challenged with SARS-CoV-2 WA1/2020 were stained with anti-CD3, anti-CD8, and an MHC class I tetramer/N_219-227_ peptide complex. Infected, unimmunized and infected, immunized controls are shown. FSC, forward scatter. (**B**) At 3 dpi, the fraction of antigen-specific (TCR^+^) CD8^+^ T cells was quantified by flow cytometry as shown by the scheme in **A** using tetramers for the N_219-227_ and S_539-546_ epitopes. The immunizing vectors are labeled below. (**C**) The weight of the spleens of immunized and challenged mice was determined 14 days postimmunization. (**D**) The fraction of antigen-specific CD8^+^ T cells in the immunized mice that expressed IFN-γ and perforin was quantified by flow cytometry. (**E**) The fraction of total CD8^+^ T cells in the immunized mice that expressed CD107a and IL-2 was quantified by flow cytometry. (**F**) Naive, effector, and central memory T cells in the immunized mice were quantified. The results are summarized in the pie charts on the right. The numbers in the control pie chart (top) show the percentage of naive, effector, and central memory cells from uninfected, unimmunized and CD40L-immunized mice. The numbers in the immunized pie chart (bottom) show the percentage of naive, effector, and central memory T cells from CD40L-N_105-113_–, CD40L-N_219-227_–, CD40L-ORF1_1637-1646_–, CD40L-S_539-546_–, and CD40L-N_219-227_- S_539-546_–immunized mice. Statistical significance was determined by Kruskal-Wallis test with post hoc Dunn’s test. Confidence intervals are shown as the mean ± SD. (*P ≤ 0.05, **P ≤ 0.01, ***P ≤ 0.001, ****P ≤ 0.001.) The experiment was done 3 times with similar results.

To determine the number of T cells to the respective epitopes generated by the vaccine, we quantified the fraction of antigen-specific CD8^+^ T cells as measured by class I MHC tetramer binding. In mice immunized with the N_219-227_ epitope vector–transduced DCs and then infected with SARS-CoV-2, about 5% of the CD8^+^ T cells were specific for the epitope (Figure 2, A and B). Infected but unimmunized mice had about 2.5% antigen-specific CD8^+^ T cells, as did uninfected/unimmunized mice, indicating that replicating SARS-CoV-2 had little effect on the number of responding CD8^+^ T cells in the spleen in the first 3 days after infection. In mice that were immunized with the CD40L-N_219-227_ vector but uninfected, the number of antigen-specific CD8^+^ T cells increased to about 7%. IFN-γ and IL-10 secretion levels also significantly increased, showing that it was the vaccination that induced the T cell response and not SARS-CoV-2 infection (Supplemental Figure 1; supplemental material available online with this article; https://doi.org/10.1172/jci.insight.167306DS1). Immunization with the dual N_219-227_ and S_539-546_ epitope vectors resulted in about 3.8% of N_219-227_-specific CD8^+^ T cells (Figure 2, A and B). Immunization with the S_539-546_ epitope–expressing vector resulted in a stronger response, with about 17% of the T cells S_539-546_ epitope specific. Infected, unimmunized mice had about 9% S_539-546_ epitope–specific CD8^+^ T cells. The dual epitope vector–transduced DCs induced a 12% S_539-546_ epitope–specific CD8^+^ T cells. The strength of the T cell response to the vaccine was evident by the splenomegaly that occurred following vaccination in which spleens increased in weight (Figure 2C). The increase was due to vaccination and not SARS-CoV-2 infection or vector-expressed CD40L as neither infection or control vector increased spleen weight. To determine the functionality of the responding T cells, we analyzed antigen-specific T cells for their expression of IFN-γ and perforin. The results showed that N_219-227_ epitope vector-transduced DCs induced the highest proportion of antigen-specific T cells expressing IFN-γ (17%) and perforin (22%) (Figure 2D). It was not possible to analyze T cells specific for the N_105-113_ and ORF1_1637-1646_ epitopes as tetramers were not available. Analysis of total CD8^+^ T cells for CD107a, marker of recent cytotoxic T lymphocyte (CTL) degranulation, showed that all the vectors increased the total proportion of positive cells and that the increase was greatest for the ORF1_1637-1646_ vector (Figure 2E). Analysis of IL-2 expression in the CD8^+^ T cells showed that all the vectors induced 2- to 4-fold increase in the proportion of IL-2^+^CD8^+^ T cells, with the largest increases induced by the N_105-113_, ORF1_1637-1646_, and S_539-546_ vectors (Figure 2E). Analysis of the memory T cell subpopulations showed that the vaccinations caused 1.5-fold decrease in the proportion of naive (CD62L^hi^CD44^lo^) cells and 1.5-fold increase in the proportion of effector cells (CD62L^lo^CD44^hi^), consistent with a high level of functional T cells (Figure 2F). The proportion of central memory cells (CD62L^hi^CD44^hi^) was slightly (1.2-fold) decreased, most likely because long-term memory had not yet been established.

### CD40L expression by vaccine vector enhances the T cell response.

**Figure 3:**
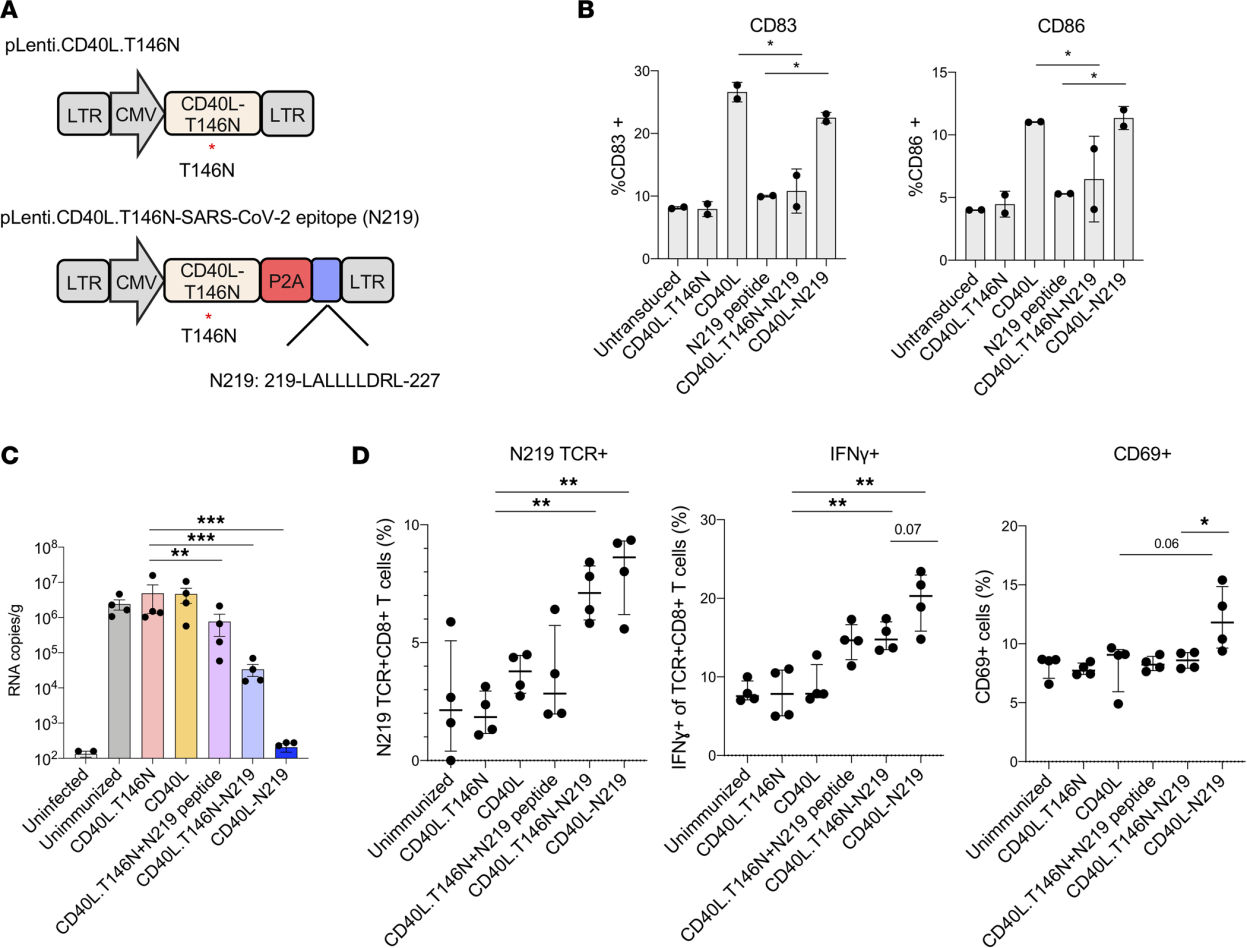
Enhancement of the antiviral response by CD40L. (**A**) The schematic diagram of lentiviral vectors expressing nonfunctional CD40L. The red asterisk indicates the T146N mutation in CD40L. (**B**) SAMHD1-KO BMDCs were transduced (MOI = 5) with lentiviral vectors expressing CD40L, mutated CD40L, CD40L-N_219-227_, and CD40L.T146N-N_219-227_ virus. After 3 days, the cells were analyzed for CD83 and CD86 by flow cytometry. (**C**) Mice were injected twice with transduced BMDCs and challenged with SARS-CoV-2 WA1/2020. After 3 days, virus load in the lung was measured by RT-qPCR. (**D**) Antigen-specific CD8^+^ T cells, IFN-γ in TCR^+^CD8^+^ T cells, and CD69^+^ cells in the immunized and challenged mice were quantified by flow cytometry. Statistical significance was determined by Kruskal-Wallis test with post hoc Dunn’s test. Confidence intervals are shown as the mean ± SD. (*P ≤ 0.05, **P ≤ 0.01, ***P ≤ 0.001.) The experiment was done twice with similar results.

The engagement of CD40 by CD40L on DCs induces their maturation and activation. Inclusion of CD40L in the vectors was intended to promote the activation and expansion of interacting antigen-specific T cells. To test whether this was the case, we constructed a control vector (pLenti.CD40L.T146N) that expressed CD40L containing an inactivating T146N mutation. To determine the functionality of the vector-encoded CD40L, we transduced DCs with vectors expressing CD40L or CD40L.T146N, with or without the N_219-227_ peptide epitope. As a control, the DCs were pulsed with synthetic N_219-227_ peptide epitope (Figure 3A). The effect of the treatments on DC maturation was determined by analyses of CD83 and CD86 expression levels. The results showed that transduction with the CD40L.T146N vectors had no effect on the baseline 8% CD83^+^ DCs and that the synthetic peptide also had no effect. In contrast, transduction with the CD40L-expressing vectors increased the percentage of CD83^+^ cells to 28% and 22%, respectively, approximating the percentage of DCs that had been transduced. The vectors also induced a similar increase in CD86 expression, though to a lesser extent (11% for both vectors) (Figure 3B). The results verified the ability of the vector-encoded CD40L to induce DC maturation.

To test the effect of CD40L on vaccine effectiveness, mice were immunized with DCs transduced with vectors expressing CD40L or CD40L.T146N, with or without N_219-227_ peptide epitope, and then challenged with SARS-CoV-2 WA1/2020. Measurement of viral load 3 dpi showed that DCs transduced with vectors expressing CD40L or CD40L.T146N alone had no effect (Figure 3C). Immunization with DCs transduced with CD40L.T146N and pulsed with synthetic N_219-227_ peptide caused 8-fold decrease in virus load. Immunization with vector expressing CD40L.T146N and N_219-227_ caused 39-fold decrease in virus load while vector expressing wild-type CD40L and N_219-227_ decreased the virus load 1,100-fold, a level indistinguishable from background.

To understand the basis of the protective T cell response generated by the vectors, we measured the number of antigen-specific CD8^+^ T cells induced in the immunized and challenged mice and evaluated their functionality as reflected by IFN-γ production, and activation state, as measured by CD69 expression. Immunization with DCs transduced with CD40L vector caused a slight increase in the number of N_219-227_-specific CD8^+^ T cells as compared with the control CD40L.T146N vector (Figure 3D). DCs expressing CD40L.T146N and pulsed with N_219-227_ peptide also slightly increased the number of antigen-specific CD8^+^ T cells. Neither of the increases were statistically significant. In contrast, immunization with vectors expressing N_219-227_ peptide and CD40L.T146N or wild-type CD40L caused a similar increase in the number of antigen-specific CD8^+^ T cells. The similar extent of antigen-specific CD8^+^ T cell proliferation induced by the mutated and wild-type CD40L was unexpected given the stronger antiviral effect of vectors expressing wild-type CD40L. Analysis of IFN-γ showed that vectors expressing CD40L or CD40L.T146N alone had no effect on the percentage of antigen-specific IFN-γ^+^ cells (Figure 3D). N_219-227_ peptide–pulsed, CD40L.T146N vector–transduced DCs caused a small increase in the number of antigen-specific IFN-γ T cells, as did the CD40L.T146N-N_219-227_ vector, though the increases were not statistically significant. The CD40L-N_219-227_ vector caused a significantly greater increase in the percentage of IFN-γ antigen–specific CD8^+^ T cells. In addition, the CD40L-N_219-227_ vector was the only one that caused an overall increase in the percentage of CD69^+^CD8^+^ T cells (Figure 3D). Taken together, the results suggest that CD40L did not increase the number of antigen-specific CD8^+^ T cells but increased the functionality and activation state of the responding CD8^+^ T cells, effects that may account for the increased protective response of the vector.

### The protective response is mediated by CD8^+^ T cells.

**Figure 4:**
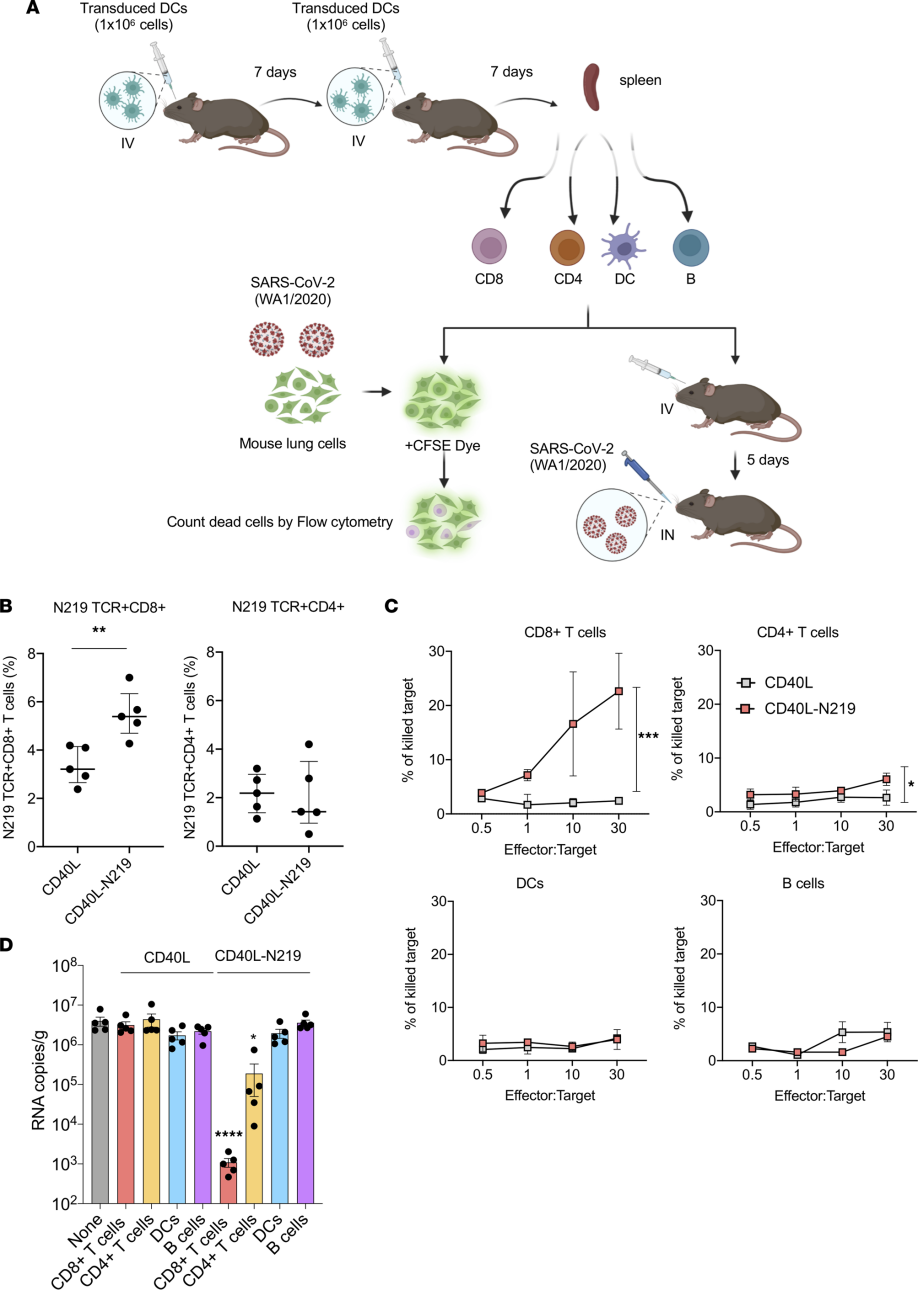
Vaccine-induced protection from infection is mediated by CD8^+^ T cells. (**A**) The cytolytic and antiviral activity of different cell types induced by vaccination was analyzed by the diagrammed experimental scheme. Mice were immunized twice with 1 × 10^6^ DCs transduced with CD40L or CD40L-N_219-227_ vector. At 7 days after second immunization, splenic CD8^+^ T cell, CD4^+^ T cell, DC, and B cell populations were isolated on magnetic beads. A portion of the cells was analyzed in an in vitro cytolytic assay in which the cells were mixed with the CFSE-labeled SARS-CoV-2–infected naive lung cells of hACE2-KI mice and the number of lysed cells was quantified by flow cytometry. A portion of each population was reinfused into recipient mice (*n* = 5), which were challenged 5 days later with SARS-CoV-2 WA1/2020. (**B**) Seven days after the second immunization, the fraction of antigen-specific CD8^+^ and CD4^+^ T cells in splenocytes was quantified by flow cytometry using tetramers for the N_219-227_ epitopes. (**C**) Cytolytic activity of the CD4^+^ T cells, CD8^+^ T cells, DCs, and B cells of control CD40L alone and CD40L-N_219-227_ vector–immunized mice was analyzed with the assay diagrammed in **A** using SARS-CoV-2–infected primary mouse lung epithelial cell targets. (**D**) Splenic CD8^+^ T cell, CD4^+^ T cell, DC, and B cell populations from immunized mice were reinfused into recipient mice (*n* = 5). At 5 days postinjection, mice were challenged with SARS-CoV-2 WA1/2020. Subgenomic viral RNA in the lungs of the mice was quantified 3 dpi. Statistical significance was determined by Kruskal-Wallis test with post hoc Dunn’s test. Confidence intervals are shown as the mean ± SD. (*P ≤ 0.05, **P ≤ 0.01, ***P ≤ 0.001, ****P ≤ 0.0001.) The experiment was done twice with similar results.

To determine the cell types that mediate the protective response induced by vaccination, we isolated CD8^+^ T cells, CD4^+^ T cells, B cells, and DCs from the splenocytes of the immunized mice on magnetic beads (Figure 4A). Analysis of the populations with cell type–specific antibodies by flow cytometry showed that they were highly pure (Supplemental Figure 2). Analysis of the CD8^+^ and CD4^+^ T cell populations showed that nearly 5% of the CD8^+^ T cells from mice immunized with the CD40L-N_219-227_ vector were tetramer^+^; the CD4^+^ T cells of the mice remained at baseline levels (Figure 4B). The cytolytic activity of each cell population was determined using an in vitro assay. In this assay, each cell population was mixed with CFSE-stained SARS-CoV-2–infected primary hACE2-KI lung cells, and after 24 hours, the cells were stained with viability dye and analyzed by flow cytometry. The results showed that the CD8^+^ T cells of the CD40L-N_219-227_–immunized mice were highly active (Figure 4C). The activity could be detected at a ratio as low as 1:1 effector cell to target cell (E/T). Considering that about 5% of the CD8^+^ T cells were antigen specific, this ratio corresponds to an actual E/T ratio of about 3:50. CD4^+^ T cells had a small amount of cytolytic activity, and no cytolytic activity was detected in DCs and B cells (Figure 4C).

To determine which of the cell types was sufficient to suppress virus replication in infected mice, purified cell populations from mice immunized with control CD40L or CD40L-N_219-227_ vectors were injected into recipient mice. After 5 days, the mice were challenged with SARS-CoV-2 WA1/2020, and 3 dpi, the virus load was measured. Cell populations isolated from the CD40L control–immunized mice had no significant effect on virus load. In contrast, CD8^+^ T cells from the CD40L-N_219-227_ vector–immunized mice caused 3,600-fold decrease in virus load (Figure 4D). CD4^+^ T cells from immunized mice decreased the virus load 21-fold on average while DCs and B cells had no effect. Taken together, the results show that the vaccine suppressed virus loads by CD8^+^ T cell-mediated cytolysis of infected lung cells.

### Immunization by direct injection of viral vector protects against SARS-CoV-2 infection.

**Figure 5:**
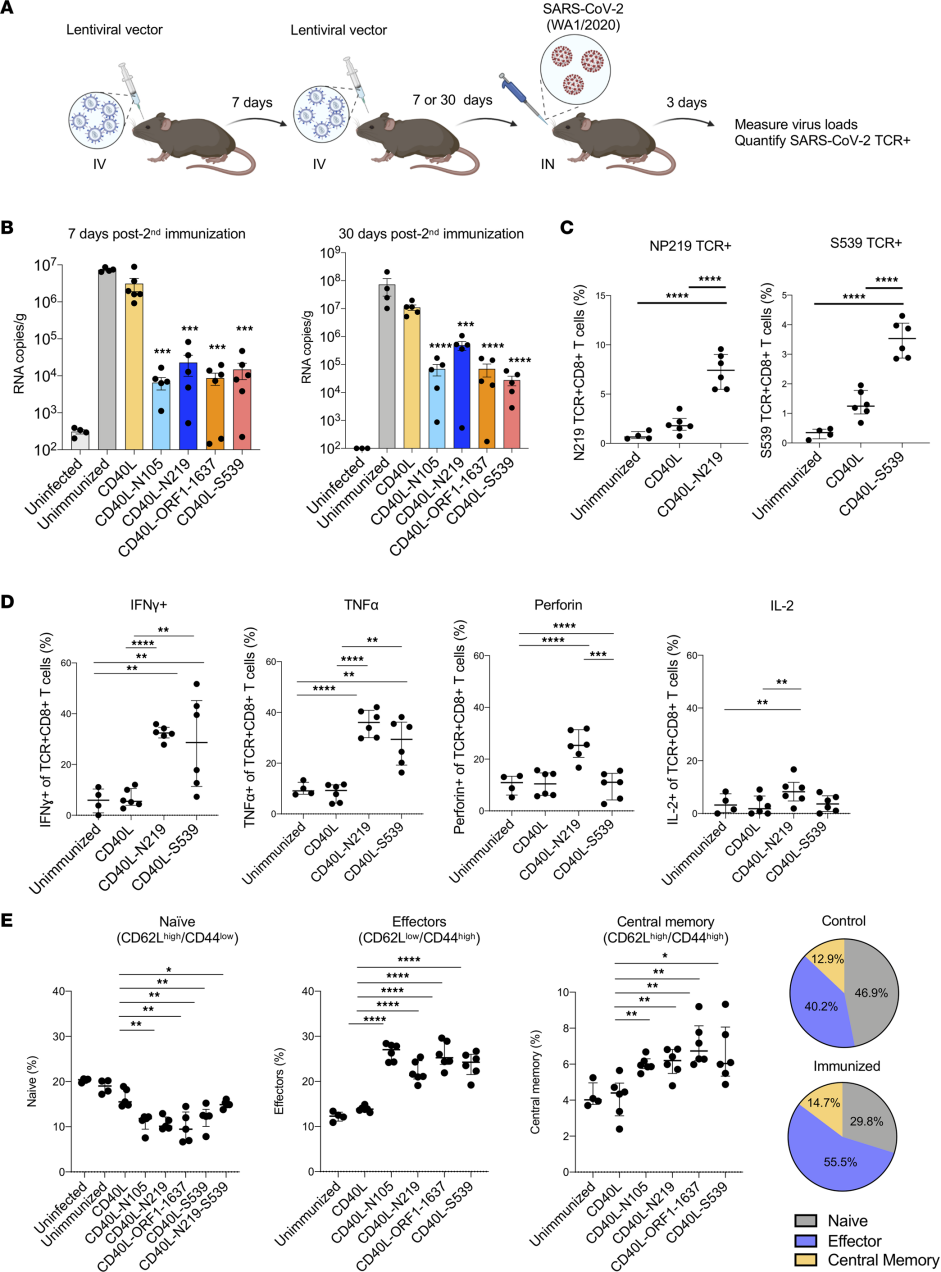
Direct lentivirus injection protects mice from SARS-CoV-2 infection. (**A**) Schematic of direct lentivirus immunization. A total of 5 × 10^6^ IU lentiviral vector encoding CD40L and T cell epitopes N_219-227_, N_105-113_, ORF1_1637-1646_, S_539-546_, and N_219-227_-S_539-546_. Lentiviral vectors were injected into hACE2-KI mice IV (*n* = 6). One week after the first immunization, the mice were re-immunized. One week or 30 days following the second immunization, the mice were challenged with 2 × 10^4^ PFU SARS-CoV-2 WA1/2020. (**B**) One week (left) or 30 days (right) following the second immunization, SARS-CoV-2 subgenomic viral RNA in the lung was quantified 3 dpi with SARS-CoV-2 WA1/2020. (**C**) Splenocytes were analyzed for the CD3^+^, CD8^+^, CD4^+^, and SARS-CoV-2–specific TCR^+^CD8^+^ T cells by flow cytometry. (**D**) IFN-γ, TNF-α, perforin, and IL-2 levels in TCR^+^CD8^+^ T cells were quantified by flow cytometry. (**E**) Naive, effector, and central memory T cells were distinguished by CD62L and CD44, then determined the population of naive (CD62L^hi^CD44^lo^), effector (CD62L^lo^CD44^hi^), and central memory cells (CD62L^hi^CD44^hi^). The results are summarized in the pie charts on the right. The percentage of naive, effector, and central memory cells of uninfected, unimmunized, and CD40L-immunized mice is shown (top). The percentage of naive, effector, and central memory T cells from CD40L-N_105-113_–, CD40L-N_219-227_–, CD40L-ORF1_1637-1646_–, CD40L-S_539-546_–, and CD40L-N_219-227_- S_539-546_–immunized mice (*P ≤ 0.05, **P ≤ 0.01, ***P ≤ 0.001, ****P ≤ 0.0001) is shown (bottom). Statistical significance was determined by Kruskal-Wallis test with post hoc Dunn’s test. Confidence intervals are shown as the mean ± SD. The experiment was done twice with similar results.

We have previously shown that a lentiviral vector–based vaccine against LCMV could be directly injected into mice, avoiding the need for ex vivo DC isolation and transduction (48). To test whether it was similarly possible to immunize against SARS-CoV-2 by direct lentivirus injection, we immunized mice with 2 IV injections of 5 × 10^6^ infectious units (IU) N_105-113_, N_219-227_, ORF1_1637-1646_, S_539-546_, N_219-227_-S_539-546_, or control CD40L vectors. After 7 days, the mice were challenged with SARS-CoV-2 WA1/2020, and virus loads were measured 3 dpi (Figure 5A). Immunization with the N_105-113_, N_219-227_, ORF1_1637-1646_, and S_539-546_ vectors suppressed virus loads 320- to 1,100-fold (Figure 5B). Challenge of the mice 30 days postimmunization showed a similar level of virus load suppression (Figure 5B). Histopathological analysis of H&E-stained lung sections showed that the lungs of control CD40L vector–immunized infected mice had signs of interstitial pneumonia with thickened alveolar septa and inflammatory cell infiltration; the lungs of mice immunized with the epitope-expressing vectors showed little sign of pneumonia with few infiltrating inflammatory cells (Supplemental Figure 3). The results showed that direct injection of the vaccine vectors provided long-term protection.

Analysis of the response to vaccination showed that immunization with the N_219-227_ vector caused the number of antigen-specific CD8^+^ T cells to increase to 7% of total CD8^+^ T cells (Figure 5C). Immunization with the S_539-546_ vector induced a similar increase. Analysis of the functionality of the responding CD8^+^ T cells showed that the antigen-specific CD8^+^ T cells were enriched in IFN-γ^+^ cells (35% for N_219-227_ vector and 30% for S_539-546_) and in TNF-α^+^ cells (32% for N_219-227_ and 30% for S_539-546_). CD8^+^ T cells responding to immunization with the N_219-227_ vector were also enriched in perforin (25%) and IL-2 (8%), suggesting that they were cytolytic (Figure 5D). Immunization with the S_539-546_ vector did not cause a significant increase, probably because fewer T cells responded to this epitope, making it more difficult to detect an increase over the background of IFN-γ^+^ cells. Analysis of the naive and memory cell populations showed that, as for immunization with transduced DCs, the vaccination resulted in 1.2-fold decreased proportion of naive (CD62L^hi^CD44^lo^) T cells, 2-fold increase in the proportion of effector T cells (CD62L^lo^CD44^hi^), and 1.5-fold increase in the proportion of central memory T cells (CD62L^hi^CD44^hi^) (Figure 5E).

### Comparison of the response to lentiviral vector and mRNA vaccines.

**Figure 6:**
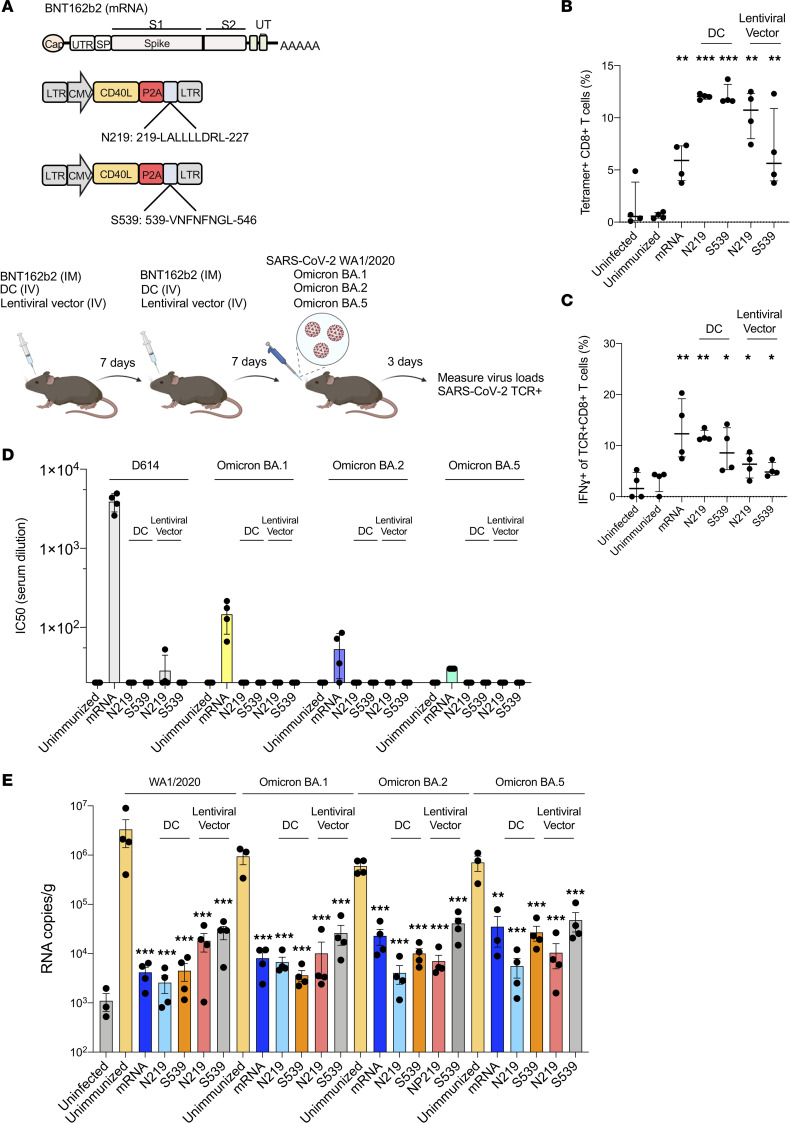
Comparison of protective efficacy of BNT162b2, lentivirus-based DC, and direct lentivirus vaccine against SARS-CoV-2 variants. (**A**) The experimental scheme is diagrammed. hACE2-KI mice were immunized with mRNA vaccine BNT162b2 (5 μg), with N_219-227_ or S_539-546_ vector–transduced DCs (1 × 10^6^), or by direct lentivirus injection of the CD40L-N_219-227_ or CD40L-S_539-546_ lentiviral vectors (*n* = 5). Uninfected and unimmunized/infected mice served as controls. All mice were boosted 7 days after the first immunization. After another 7 days, the mice were challenged by infection with SARS-CoV-2 WA1/2020 or Omicron variants. (**B**) Antigen-specific CD8^+^ T cells of the immunized and challenged mice were stained with the corresponding N_219-227_ and S_539-546_ tetramers and analyzed by flow cytometry. (**C**) The tetramer^+^IFN-γ^+^CD8^+^ T cells of the immunized mice were quantified by flow cytometry. (**D**) Sera from the immunized mice was collected 7 days postboost. Neutralizing antibody titers against viruses with D614, BA.1, BA.2, and BA.5 S were measured. The IC_50_ of the serum from each mouse is shown. (**E**) Immunized mice were challenged with SARS-CoV-2 WA1/2020 or BA.1, BA.2, BA.5. At 3 dpi (SARS-CoV-2 WA1/2020) or 2 dpi (BA.1, BA.2, and BA.5), virus load in the lungs was determined. Statistical significance was determined by Kruskal-Wallis test with post hoc Dunn’s test with confidence intervals shown as the mean ± SD. (*P ≤ 0.05, **P ≤ 0.01, ***P ≤ 0.001.) The experiment was done twice with similar results.

The highly effective mRNA vaccines produced by Pfizer and Moderna encode a modified S protein. While the vaccines induce high titers of neutralizing antibodies, the S protein contains several CD8^+^ T cell epitopes that induce a T cell response. To better understand the role of T cell responses to the mRNA and lentiviral vector vaccines, we immunized mice with BNT162b2 mRNA vaccine (IM), with lentiviral vector–transduced DCs (IV) or by direct injection with the lentiviral vectors (IV) (Figure 6A). Seven days later, the mice were boosted and, after another 7 days, challenged with SARS-CoV-2 WA1/2020 or Omicron BA.1, BA.2, or BA.5. N_219-227_- and S_539-546_-specific CD8^+^ T cells and IFN-γ^+^CD8^+^ T cells, and virus loads were quantified 3 dpi. Analysis of the antigen-specific T cells showed that the mRNA vaccine stimulated a relatively moderate level (6%) of S_539-546_-specific CD8^+^ T cells (N_219-227_ was not tested as the vaccine does not encode this epitope); transduced DCs induced the strongest response, resulting in large numbers (12%) of N_219-227_ and S_539-546_ specific CD8^+^ T cells; and direct lentiviral vector injection induced a strong response (10%) to N_219-227_ and a lesser response (5%) to S_539-546_ (Figure 6B). Intracellular expression of IFN-γ was also 5- to 10-fold upregulated in the spleen, suggesting both DC and direct lentiviral vector vaccines strengthen CTL activity (Figure 6C).

mRNA vaccination induces high titers of neutralizing antibody against the homologous S protein. In contrast, the N_219-227_ epitope vector–transduced DC does not contain S protein sequence; the S_539-546_ vector encodes only a small portion of the S protein that lies outside the ACE2 interaction region. To evaluate the neutralizing antibody response to the mRNA and lentiviral vector vaccines, we compared the serum neutralizing antibody titers of the immunized and challenged mice against viruses pseudotyped by the D614 and Omicron BA.1, BA.2, and BA.5 S proteins. The results showed that the mRNA vaccine elicited high titers of neutralizing antibodies (IC_50_ of 3,800) against the homologous D614 S protein but much lower titers against the variants (20-fold lower for BA.1 and 50-fold lower for BA.2 and 150-fold lower for BA.5). Immunization with transduced DCs or direct lentiviral vector injection did not result in neutralizing titers, verifying that the vaccine did not induce neutralizing antibodies (Figure 6D).

Because the T cell response is directed against epitopes in several of the viral proteins, it has the potential to be more effective than neutralizing antibodies, which are directed only against the highly variable S protein and therefore subject to immunoevasion. To compare the level of protection provided by the different vaccines, we immunized mice and challenged with the homologous SARS-CoV-2 WA1/2020 or Omicron BA.1, BA.2, and BA.5 variants. The results showed that the mRNA vaccine decreased virus loads 800-fold for mice infected with the homologous SARS-CoV-2 WA1/2020 compared with unimmunized controls. The N_219-227_ and S_539-546_ vector–transduced DCs decreased virus loads to a similar extent against that virus (Figure 6E). Directly injected vector decreased the virus load to a lesser extent (120- to 180-fold for N_219-227_ and S_539-546_). Similar results were obtained upon challenge with BA.1. For mice challenged with BA.2 and BA.5, the mRNA vaccine lost some of its potency, decreasing the virus load by 120- to 180-fold, while the N_219-227_ vaccination maintained its highly protective effect. Directly injected N_219-227_ vector was also highly protective against BA.2 and BA.5. The S_539-546_ vector was less effective. The results verify the strong cross-protection provided by the T cell vaccine. The lack of cross-reactivity of the antibodies elicited by the mRNA vaccine on the Omicron variants suggests that the protection was mediated by T cells and not neutralizing antibody.

## Discussion

We show here that a lentiviral vector–based vaccine for SARS-CoV-2 encoding single CD8^+^ T cell epitopes induced a high degree of protection from infection and disease in a mouse model. The protective response was rapid, protecting mice 14 days after the first dose, and was mediated by antigen-specific T cells. The vaccine did not raise neutralizing antibodies, and the protection could be adoptively transferred by the CD8^+^ T cells. The vaccine was most effective when administered by the injection of ex vivo–transduced DCs, decreasing the virus load 1,000-fold upon subsequent challenge with the homologous virus. Direct IV injection of the lentiviral vector was also protective, although the decrease in virus load was less dramatic, decreasing virus loads by 100-fold. Both routes of immunization prevented pulmonary damage and infiltration of inflammatory cells into the lungs. The vaccination induced the expansion of epitope-specific T cells that were highly functional as shown by the expression of IFN-γ, perforin, and CD107a, which are markers of active cytolytic T cells. Vectors were tested that encoded CD8^+^ T cell epitopes derived from the S, N, and ORF1. Of the 4 epitopes tested, the nucleocapsid-derived epitope N_219-227_ induced the highest level of antigen-specific polyfunctional CD8^+^ T cells. The lentiviral vaccine was equally protective against the ancestral SARS-CoV-2 WA1/2020 and Omicron BA.1, BA.2, and BA.5 variants. A previous report by Dangi et al. (50) showed that a vaccine that combined the S and N provided stronger protection than the S protein alone. In that study, an N-alone vaccine provided partial protection. It is possible that the lentiviral vector approach more strongly induced CD8^+^ T cells, either due to long-lasting antigen expression or due to the effect of CD40L on DCs in enhancing antigen presentation and T cell activation.

The protective effect of the lentiviral vector–based vaccine appeared to be the result of CD8^+^ T cells and not antibody. The N and ORF1 epitopes are not neutralizing epitopes, and the S_539-546_ epitope lies outside the RBD of the S protein. Moreover, neutralizing antibody was not detected, even postchallenge. It is possible that neutralizing antibody was produced in the vaccinated mice at later time points postchallenge but this was not tested. We also did not measure non-neutralizing antibody, but it seems unlikely that there would be significant titers of such antibodies in the absence of neutralizing antibody. In addition, adoptive transfer of CD8^+^ T cells from the vaccinated mice was sufficient to protect an unvaccinated mouse from infection. The transfer of CD4^+^ T cells resulted in a minor decrease in virus load, perhaps caused by presentation of the peptide on MHC class II. In the vaccinated and live virus–challenged mice, generally only about 40% of responding CD8^+^ T cells appeared to be highly functional as judged by cytokine production. The relatively small proportion of antigen-specific cell activation may be a function of the rapid clearance of virus, such that many cells did not encounter antigen. We found similar results upon immunization against LCMV (47, 48), suggesting that the relatively small number of activated antigen-specific T cells may be caused by the effectiveness of the vaccination in preventing virus replication.

The T cell response to SARS-CoV-2 vaccination has a wider breadth than the antibody response as T cells respond to epitopes contained within many of the viral proteins while the neutralizing antibody response is targeted only to the S protein. Consistent with this, the lentiviral vector–based T cell vaccine was equally effective against the SARS-CoV-2 WA1/2020 and Omicron variants. In contrast, the antibodies elicited in the mouse by the Pfizer/BNT162b2 mRNA vaccine barely cross-reacted on BA.1, BA.2, and BA.5. While the mRNA vaccine-elicited antibody did not cross-react on the Omicron variants, the vaccination was highly effective in decreasing virus loads in BA.1-, BA.2-, and BA.5-infected mice, suggesting that the protection was largely mediated by T cells responding to epitopes in the vaccine-encoded S protein. The Pfizer/BNT162b2 vaccine induced T cells specific for the S_539-546_ epitope but in numbers significantly smaller (Figure 6B) than those induced by the S_539-546_ lentiviral vector vaccine. The greater T cell response invoked by lentiviral vector vaccine may result from targeting the vector to DCs and other antigen-presenting cells, long-lasting antigen expression, and the effect of CD40L on promoting maturation of the DCs. The effect of CD40L on increasing vaccine effectiveness was demonstrated by the increased expression of CD83 and CD86 on the DCs and the 10-fold greater virus load suppression of vectors that encoded a functional CD40L as compared with the control vector that expressed a nonfunctional CD40L mutant.

Lentiviral vector transduction efficiency is limited in mouse and human cells by the myeloid restriction factor SAMHD1 (51–55). In this study, we circumvented the restriction through the use of SAMHD1-KO mice. SAMHD1-KO DCs were transduced more efficiently, and direct injection of the vectors results in the preferential transduction of DCs and other myeloid cells. In human cells, SAMHD1 restriction can be circumvented by generating lentiviral vectors that package the SIV accessory protein Vpx (56–59). Because Vpx fails to induce the degradation of murine SAMHD1, this strategy is not effective in mice, necessitating the use of the KO strain to mimic the transduction efficiency that can be achieved using Vpx-containing virions in humans.

While T cells do not provide sterilizing immunity, as they act only postinfection, they are very effective at decreasing virus loads. Moreover, they target viral epitopes present in several of the viral proteins in addition to the S protein to which antibody neutralization is restricted. Moreover, the neutralizing epitopes to which antibodies are targeted are under selective pressure that leads to escape in rapidly emerging variants (11–13, 60–65). Current mRNA- and viral vector–based vaccines encode only the S protein and thus induce a T cell response to only a small number of viral epitopes. The incorporation of additional T cell epitopes would be likely to increase vaccine effectiveness, making vaccines less susceptible to immunoevasion by novel variants, and, given the long half-life of T cell memory, increase vaccine durability. While this study was based on lentiviral vectors, which are not currently approved for use in humans, the principles demonstrated here are likely to hold generally for protein- and vector-based vaccines.

## Methods

### Cells.

HEK293T (ATCC) cells were cultured in DMEM/10% FBS. ACE2.TMPRSS2.Vero E6 cells (ATCC) were cultured in DMEM/10% FBS with the addition of 1 μg/mL puromycin. BMDCs were prepared by extracting bone marrow cells from the hind legs of 6- to 10-week-old female SAMHD1-KO mice. The cells (5 × 10^6^ cells) were differentiated in a 15 cm Petri dish in RPMI/10% FBS, sodium pyruvate, 2 mM l-glutamine, and 50 μM 2-mercaptoethanol containing 10 ng/mL murine GM-CSF (PeproTech). The media were replenished on days 3 and 6, and the nonadherent cells were harvested on day 8.

### Mice.

C57BL/6 mice were obtained from Taconic. hACE2-KI mice [B6.129S2(Cg)-Ace2tm1(ACE2)Dwnt/J] were obtained from The Jackson Laboratory. SAMHD1-KO mice were provided by Axel Roers at the Technische Universität Dresden, Dresden, Germany (66). Animal use and care were approved by the NYU Langone Health Institutional Animal Care and Use Committee (approval 170304) according to the standards set by the Animal Welfare Act.

### Plasmids.

The lentiviral expression vectors pLenti.CD40L have been previously described (47, 49). pMDL, Rev, and pLenti.GFP.NLuc to generate pseudotyped virus have been previously described (67). To construct pLenti.CD40L-N_105-113_, -N_219-227_, -ORF1_1637-1646_, S_539-546_, and -N_219-227_-S_539-546_, CD40L was fused to the picornavirus P2A sequence and N_105-113_ (SPRWYFYYL) or N_219-227_ (LALLLLDRL) or ORF1_1637-1646_ (TTDPSFLGRY) or S_539-546_ (VNFNFNGL) by overlap extension PCR (40). The amplicon was cleaved with Xba-I and Sal-I and ligated to similarly cleaved pLenti.CMV.GFP.puro (Addgene), removing the GFP gene. To generate pLenti.CD40L.T146N, the inactivating point mutation T146N was introduced into CD40L by overlap extension PCR with the primers containing 5′-Xba-I and 3′-Sal-I sites. The amplicon was then subcloned into pLenti.CMV.GFP.puro.

### Lentiviral vector stock preparation.

Lentiviral vector stocks were generated by calcium phosphate cotransfection of HEK293T cells with pMDL (provided by Luigi Naldini, Salk Institute, La Jolla, California, USA), pcRev (provided by Thomas Hope, Salk Institute, La Jolla, California, USA), pcVSV-G, and pLenti (both made in-house) constructs at a ratio of 28:10:7:2 as previously described (67). Virus-containing supernatant was harvested 2 dpi, filtered at 0.45 μm, and stored at –80°C. The infectious titer was determined on HEK293T cells by flow cytometry as the number of GFP^+^ or CD40L^+^ cells/mL.

### SARS-CoV-2 stock preparation.

Stocks of SARS-CoV-2 WA1/2020 (BEI Resources, NR-52281) were prepared by infection of Vero E6 cells at MOI = 0.01. Omicron BA.1, BA.2 (BEI Resources, NR-56781), and BA.5 virus stocks (BEI Resources, NR-58616) were prepared by infection of ACE2-TMPRSS2 Vero cells (from Meike Dittmann and Bruno Rodriguez-Rodriguez, NYU Grossman School of Medicine) at MOI = 0.01. The medium was replaced 2 hours postinfection to remove the input virus. Three dpi (SARS-CoV-2 WA1/2020) and 5 dpi (Omicron BA.1, BA.2, BA.5), the virus-containing supernatant was harvested and filtered through a 0.45 μm filter, and the virus was concentrated on an Amicon Ultra Filter Unit by centrifugation at 3,000*g* for 15 minutes at 4°C and stored in aliquots at –80°C. The virus was titered by plaque assay on ACE2.TMPRSS2.Vero E6 cells.

### Flow cytometry.

Mouse splenocytes were blocked with anti-CD16/CD32 mAbs and stained with eFluor 450 viability dye (93: catalog 101320, BioLegend). Antibodies used for cell surface proteins were Alexa Fluor 700–anti-CD3 (17A2: catalog 100215), PerCP-Cy5.5–anti-CD8a (57-6.7: catalog 100734), APC-Cy7–anti-CD4 (GKq.5: catalog 100412), PE-Cy7–anti-CD19 (6D5: catalog 115520), PE–anti-CD62L (W18021D: catalog 161203), PE-Cy7–anti-CD44 (IM4: catalog 103024) (all BioLegend), and APC–anti-CD11c (3.9: catalog 17-0116-42, Thermo Fisher Scientific). The cells were then stained with fluorescence antibodies or H-2D(b) SARS-CoV-2 N_219-227_ LALLLLDRL (Alexa Fluor 647–Labeled Tetramer) or H-2K(b) SARS-CoV-2 S_539-546_ VNFNFNGL (Alexa Fluor 647–Labeled Tetramer) (NIH Tetramer Facility, Emory University). For intracellular staining, the cells were permeabilized in PBS containing 0.1% saponin and fixed for 10 minutes in 4% paraformaldehyde. The cells were then stained with PE–anti–IFN-γ (XMG1.2: catalog 505808), APC-cy7–anti–TNF-α (MP6-XT22: catalog 506343), PE–anti-perforin (S16009A: catalog 154305), and PE-cy7–anti-granzyme (QA16A02: catalog 372213) (BioLegend). The cells were analyzed on an LSR II flow cytometer (BD) and the data were analyzed with FlowJo software.

### Cytokine concentrations.

Splenocytes from immunized mice were placed in 96-well culture dishes (1 × 10^6^ cells/well) and incubated with or without synthetic peptide epitope (1 μg/mL) for 16 hours at 37°C. Culture supernatant (10 μL) was then harvested to measure the concentrations of IFN-γ and IL-10 by cytokine bead array using a BD Cytometric Bead Array Mouse Inflammation Kit (BD Biosciences).

### Immunization.

For DC vaccination, SAMHD1-KO BMDCs (5 × 10^6^ cells) were plated in a 10 cm Petri dish and transduced with lentiviral vector at MOI = 5 for 16 hours. hACE2-KI mice were injected IV with 1 × 10^6^ transduced BMDCs. After 7 days, the mice were re-immunized. For direct lentivirus immunization, 5 × 10^6^ IU lentivirus was injected IV. After 7 days, the mice were re-immunized. For BNT162b2 vaccine, 50 μL (5 μg) of BNT162b2 was injected IM (68, 69). After 7 days, the mice were re-immunized. Seven days after the second immunization, the mice were challenged with 2 × 10^4^ PFU of SARS-CoV-2 WA1/2020 and Omicron BA.1, BA.2, and BA.5 by IN instillation.

### Virus load.

SARS-CoV-2 subgenomic E RNA levels were measured by RT-qPCR with TaqMan probe (Applied Biosystems). RNA was prepared from 200 μL homogenized tissue using the *Quick*-RNA Miniprep kit (Zymo Research). Equal amount of lung RNA was mixed with TaqMan Fast Virus 1-step Master Mix (Applied Biosystems), 10 mM forward and reverse primers, and 2 mM probe. PCR was for 5 minutes at 50°C followed by 95°C/20 seconds and 40 cycles 95°C/3 seconds, 60°C/30 seconds). Subgenomic E gene were detected by using forward primer subgenomic F (CGATCTCTTGTAGATCTGTTCTC), reverse primer E_Sarbeco_R/2019-nCoV_N1-R), and probe (E_Sarbeco_P1/2019-nCoV_N1-P). Mouse GAPDH was detected by using mGAPDH.forward (CAATGTGTCCGTCGTGGATCT), mGAPDH.reverse (GTCCTCAGTGTAGCCCAAGATG), and mGAPDH probe (CGTGCCGCCTGGAGAAACCTGCC). SARS-CoV-2 standard plasmids were generated for transcription by commercial positive plasmid (2019-nCoV_E Positive Control, IDT: 10006896) transcribed in vitro. A standard curve was generated using 10-fold dilutions of SARS-CoV-2 RNA standard produced by in vitro transcription.

### Adoptive transfer.

hACE2-KI mice were immunized by IV injection of 1 × 10^6^ cells of N_219-227_ lentiviral vector–transduced BMDCs. One week postimmunization, mice were re-immunized with 1 × 10^6^ cells of transduced BMDCs. At 1 week after the second immunization, mice were sacrificed and the spleens were harvested. CD8^+^ T cells, CD4^+^ T cells, B cells, and DCs were isolated on CD8, CD4, CD19, and CD11c Microbeads UltraPure (Miltenyi Biotec), respectively. A total of 1 × 10^6^ of each cell type were injected into mice via IV injection. Five days postinjection, the mice were challenged with 2 × 10^4^ PFU of SARS-CoV-2 WA1/2020. On 3 dpi, virus load in lung was measured by RT-qPCR.

### CTL assay.

Whole lung tissue from hACE2-KI mice was disaggregated by passage through a 100 μm mesh in 5 mL ACK lysing buffer. After incubation for 5 minutes at room temperature, the cells were treated for 30 minutes with 1.5 mg/mL collagenase (Thermo Fisher Scientific) and 0.1 mg/mL DNase (MilliporeSigma). The cells were then passaged again through a 100 μm mesh, mixed with 10 mL RPMI/10% FBS, pelleted by centrifugation for 5 minutes at 375*g*, and resuspended in 10 mL in medium. The cells (1 × 10^5^) were then infected with SARS-CoV-2 WA1/2020 at MOI = 0.5. The following day, the cells were labeled with 5 μM CFSE in serum-free medium for 15 minutes at 37°C. The reaction was quenched by the addition of 10 mL RPMI/10% FCS. The cells were then used as targets in the CTL assay. Isolated effector cells from immunized mice were added on 2 × 10^4^ target cells at different ratios (30:1, 10:1, 1:1, and 0.5:1) and incubated for 24 hours. The cells were stained with Fixable Viability Dye eFluor 450 (Thermo Fisher Scientific) and dead cells in target cells were quantified by flow cytometry on an LSR II.The data were analyzed with FlowJo software.

### Histology.

Tissues were immersion-fixed in 10% neutral buffered formalin for 72 hours at room temperature and then processed through graded ethanol and xylene and into paraffin in a Leica Peloris automated processor. Five-micrometer, paraffin-embedded sections were deparaffinized and stained with hematoxylin (Leica, 3801575) and eosin (Leica, 3801619) on a Leica ST5020 automated histochemical strainer. Slides were scanned at original magnification 40× on a Leica AT2 whole-slide scanner and images transferred to the NYU Omero web-accessible image database.

### SARS-CoV-2 S protein lentiviral pseudotype assay.

Lentiviruses pseudotyped by D614 and Omicron S protein were produced by cotransfection of HEK293T cells with pMDL, plenti.GFP.NLuc, and S protein expression vectors encoding 19 amino acid cytoplasmic tail deletions, as previously reported (67). Supernatants were harvested 2 days posttransfection and concentrated by ultracentrifugation at 30,000*g*, 90 minutes, 4°C. The viruses were normalized for reverse transcriptase by an RT-qPCR–based assay using the reported primers and TaqMan probe (70). RT-qPCR data were converted to absolute mass (pg/mL) using a standard curve generated with pure HIV-1 reverse transcriptase. For the lentiviral pseudotype assay, the virus was incubated with serially diluted mouse sera for 30 minutes. The treated virus was added to ACE2.HEK293T cells, and after 2 days, infectivity was measured by NanoGlo assay (Nanolight).

### Statistics.

Statistical significance was determined by Kruskal-Wallis test with post hoc Dunn’s test. Significance (*P* < 0.05) was calculated based on 2-sided testing and is shown in the figures as the mean ± SD.

### Study approval.

Animal procedures were performed with the written approval of the NYU Animal Research Committee (approval 170304) in accordance with all federal, state, and local guidelines.

## Author contributions

TT and NRL designed the experiments. TT, JYP, and BMD carried out the experiments. TT, JYP, and NRL wrote the manuscript.

## Supplementary Material

Supplemental data

## Version 1 04/10/2023

Electronic publication
