# Supplementary material for: Single-epitope T cell–based vaccine protects against SARS-CoV-2 infection in a preclinical animal model
Source: JCI Insight. 2023 Apr 10;8(7):e167306. doi: 10.1172/jci.insight.167306 (PMC10132166; doi:10.1172/jci.insight.167306)
Supplement: Supplemental data [file jciinsight-8-167306-s049.pdf]

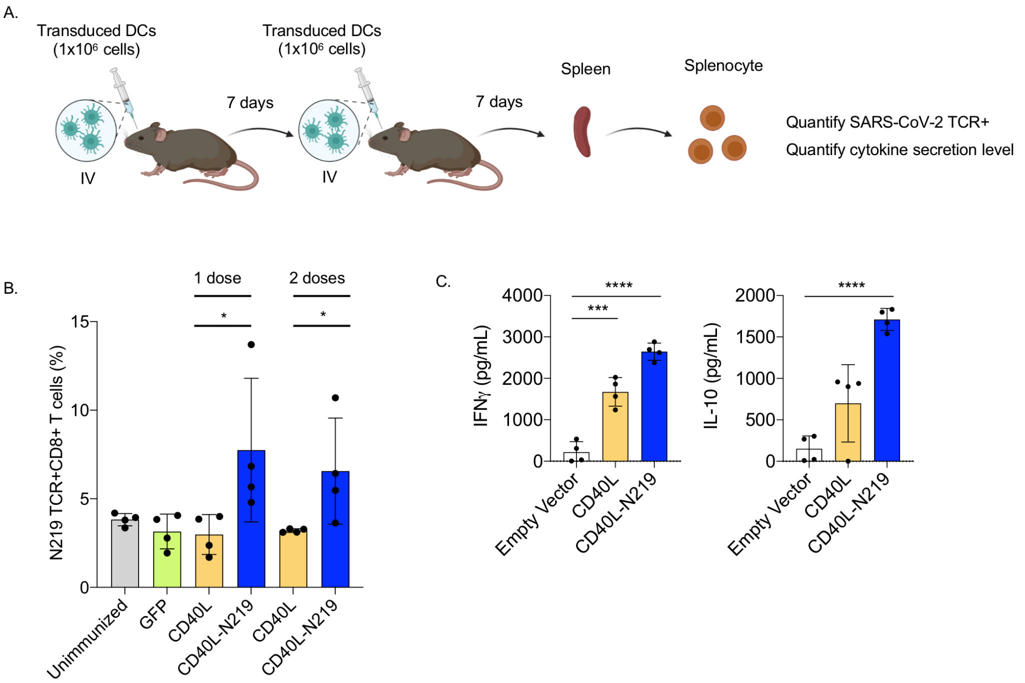

Supplementary Figure 1. Second doses immunization by transduced DC induced a strong immune response.

(A) Schematic of immunization. DCs were transduced with lentivirus encoding GFP, CD40L, and CD40L-$N_{219-227}$. $1x10^6$ cells of transduced DCs were injected into ACE2 transgenic mice via IV injection (n=4). One week after first immunization, mice were immunized again with $1x10^6$ cells of transduced DCs.

(B) After first and second doses of DC injection, splenocytes were harvested from immunized mice. The numbers of SARS-CoV-2 TCR+ CD8+ T cells were determined by flow cytometry.

(C) Seven days post-second immunization, splenocytes were incubated with 1 μg/ml of $N_{219-227}$ peptide for 3 days after which cytokine levels (IFNγ and IL-10) in the supernatant were determined by cytokine bead array. Statistical significance was determined by Kruskal-Wallis test with post hoc Dunn's test. Confidence intervals are shown as the mean ± SD. (*P ≤ 0.05, ***P ≤ 0.001, ****P ≤ 0.0001). The experiment was done twice with similar results.

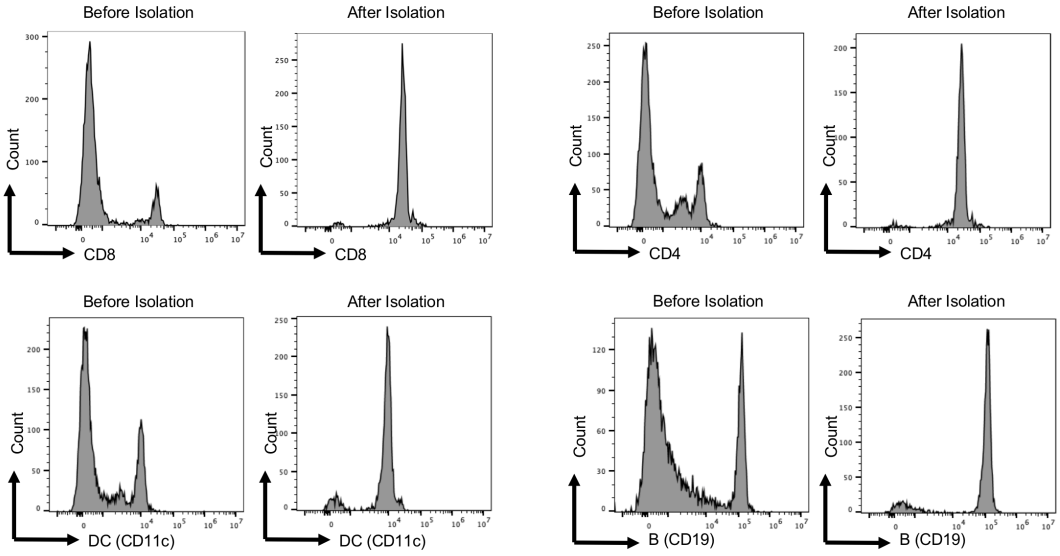

Supplementary Figure 2. Analysis of magnetic bead-purified cell populations.

Mice were immunized twice with DCs transduced with CD40L or CD40L-$N_{219\text{-}227}$ vector. Seven days post-second immunization, splenic CD8+ T, CD4+ T, DC, and B cell populations were isolated on magnetic beads. The populations were analyzed by flow cytometry with antibodies to CD8, CD4, CD11c, and CD19. The experiment was done twice with similar results.

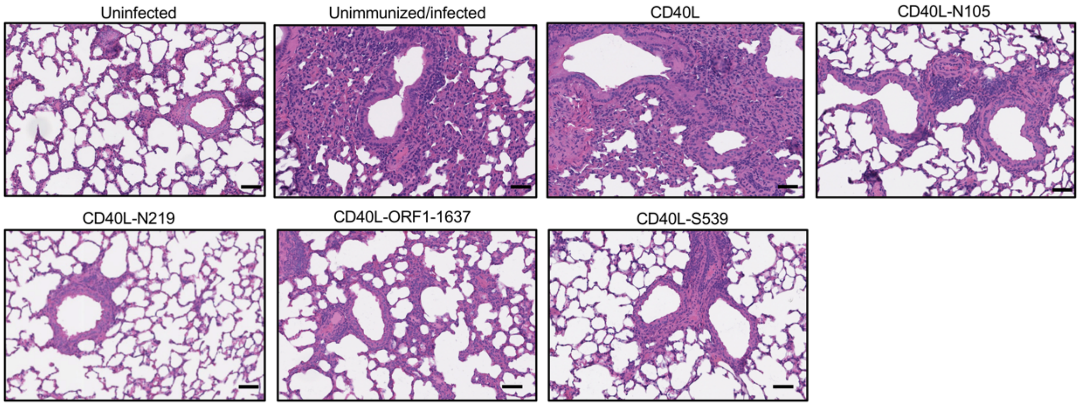

Supplementary Figure 3. Histology of lungs from mice vaccinated by direct lentivirus injection.

Hematoxylin and eosin stained lung sections of mice immunized with direct lentivirus injection and challenged with SARS-CoV-2 WA1/2020. 50μm scale bars are at lower right.
